# Supplementary material for: Serum Exosomal MicroRNA, miR-10b-5p, as a Potential Diagnostic Biomarker for Early-Stage Hepatocellular Carcinoma
Source: J Clin Med. 2020 Jan 20;9(1):281. doi: 10.3390/jcm9010281 (PMC7019940; doi:10.3390/jcm9010281)
Supplement: Supplementary file 1 [file jcm-09-00281-s001.pdf]

Table S1. miR primer sequences used in the present study.

microRNA	Primer sequence
has-miR-10b-5p	5'-TAC CCT GTA GAA CCG AAT TTG TG-3'
has-miR-18a-5p	5'-TAA GGT GCA TCT AGT GCA GAT AG-3'
has-miR-215-5p	5'-ATG ACC TAT GAA TTG ACA GAC-3'
has-miR-940	5'-AAG GCA GGG CCC CCG CTC CCC-3'
has-miR-1228-3p	5'-TCA CAC CTG CCT CGC CCC CC-3'

miR, microRNA.

Table 2. List of predominantly overexpressed miRs in HCC-derived exosomes and tumor tissues of patients with HCC in TCGA database.

microRNAs	Hep3B_FC	Huh-7_FC	TCGA_Tumor_FC	P value
has-miR-940	1.77	1.63	1.38	$1.26X10^{-13}$
has-miR-301b-3p	2.38	1.99	1.56	$6.89X10^{-26}$
has-miR-135a-5p	4.64	1.56	1.98	$1.3X10^{-29}$
has-miR-618	4.95	1.81	1.45	$6.83X10^{-7}$
has-miR-766-3p	6.20	2.44	1.65	$6.43X10^{-17}$
has-miR-551b-3p	5.78	2.65	1.34	$3.76X10^{-3}$
has-miR-19b-1-5p	9.13	1.81	1.16	$3.33X10^{-2}$
has-miR-196a-5p	1.76	23.82	1.60	$5.21X10^{-11}$
has-miR-96-5p	13.45	3.48	4.15	$2.73X10^{-55}$
has-miR-18a-5p	20.86	2.38	2.52	$1.92X10^{-28}$
has-miR-17-5p	74.22	1.72	1.88	$3.09X10^{-13}$
has-miR-182-5p	42.60	3.45	4.61	$7.47X10^{-33}$
has-miR-183-5p	56.54	3.58	5.53	$1.97X10^{-32}$
has-miR-10b-5p	101.32	12.47	8.75	$7.15X10^{-35}$
has-miR-196b-5p	180.07	9.71	2.65	$9.60X10^{-22}$
has-miR-1269a	137.69	91.38	11.97	$3.03X10^{-7}$
has-miR-1269b	227.03	190.79	5.33	$8.47X10^{-17}$
has-miR-215-5p	4475.03	101.77	2.42	$2.34X10^{-32}$

miR, microRNA; TCGA, The Cancer Genomic Atlas database; FC, fold change.

Table 3. Analysis of diagnosing HCC using combination of serum exosomal miR-10b-5p and serum AFP.

	P vs AFP	AUC	95% CI	Sensitivity (%)	Specificity (%)	PPV (%)	NPV (%)
**HCC vs Non tumor**							
	***P* vs AFP**	**AUC**	**95% CI**	**Sensitivity (%)**	**Specificity (%)**	**PPV (%)**	NPV (%)
AFP (20 ng/ml)	1	0.707	0.635-0.773	37.778	71.591	57.627	52.941
miR-10b-5p (1.8 fold)	<0.0001	0.932	0.884-0.964	91.111	75.000	78.846	89.189
AFP+ miR-10b-5p	<0.0001	0.944	0.899-0.973	90.000	84.500	88.043	89.535
**mUICC I&II vs Non tumor**							
	P vs AFP	AUC	95% CI	Sensitivity (%)	Specificity (%)	PPV (%)	NPV (%)
AFP (20 ng/ml)	1	0.597	0.509-0.681	15.217	71.591	21.875	61.765
miR-10b-5p (1.8 fold)	<0.0001	0.946	0.894-0.978	93.478	75.000	66.154	95.652
AFP+ miR-10b-5p	<0.0001	0.946	0.893-0.978	86.957	93.182	86.957	93.182
**mUICC I vs Non tumor**							
	P vs AFP	AUC	95% CI	Sensitivity (%)	Specificity (%)	PPV (%)	NPV (%)
AFP (20 ng/ml)	1	0.542	0.449-0.634	9.375	71.591	10.714	68.478
miR-10b-5p (1.8 fold)	<0.0001	0.934	0.874-0.971	90.652	75.000	56.863	95.652
AFP+ miR-10b-5p	<0.0001	0.933	0.872-0.970	84.375	88.636	72.973	93.976
**HCC vs CHB&LC**							
	P vs AFP	AUC	95% CI	Sensitivity (%)	Specificity (%)	PPV (%)	NPV (%)
AFP (20 ng/ml)	1	0.597	0.514-0.676	37.778	58.333	57.627	38.462
miR-10b-5p (1.8 fold)	<0.0001	0.931	0.878-0.966	91.111	78.333	86.316	85.455
AFP+ miR-10b-5p	<0.0001	0.938	0.886-0.971	92.222	85.000	90.217	87.931
**mUICC I&II vs CHB&LC**							
	P vs AFP	AUC	95% CI	Sensitivity (%)	Specificity (%)	PPV (%)	NPV (%)
AFP (20 ng/ml)	1	0.545	0.445-0.642	15.217	58.333	21.875	47.297
miR-10b-5p (1.8 fold)	<0.0001	0.946	0.884-0.981	93.478	78.333	76.786	94.000
AFP+ miR-10b-5p	<0.0001	0.945	0.883-0.980	86.957	93.333	90.909	90.323
**mUICC I vs CHB&LC**							
	P vs AFP	AUC	95% CI	Sensitivity (%)	Specificity (%)	PPV (%)	NPV (%)
AFP (20 ng/ml)	1	0.608	0.500-0.708	9.375	58.333	10.714	54.688
miR-10b-5p (1.8 fold)	<0.0001	0.935	0.864-0.976	90.625	78.333	69.048	94.000
AFP+ miR-10b-5p	<0.0001	0.923	0.849-0.969	90.625	85.000	76.316	94.444

Supporting Figure 1

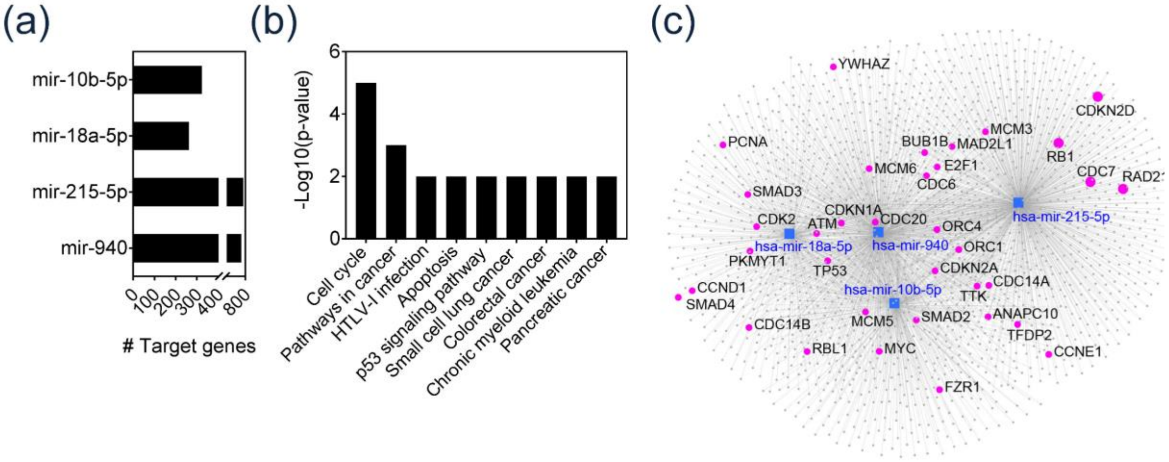

Figure S1. Prediction of target genes corresponding to each exo-miR using miRanda database.

Supporting Figure 4

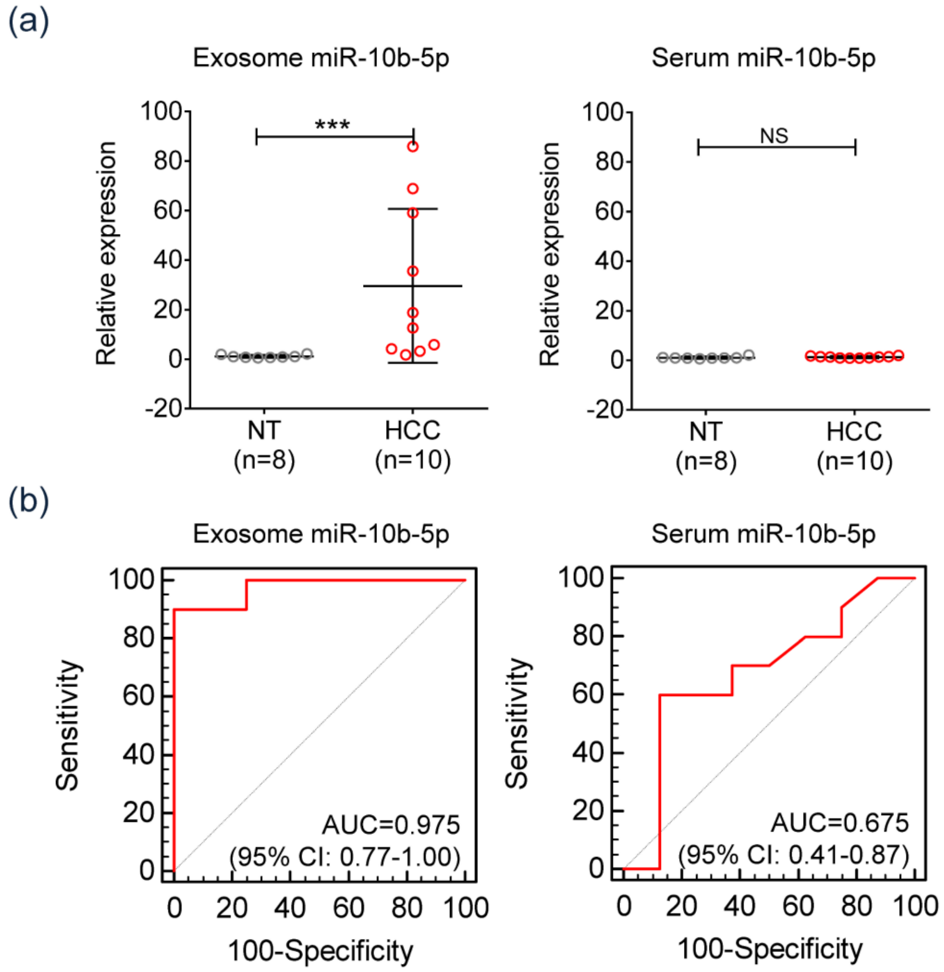

Figure S2. Comparison between diagnostic efficiencies of serum exo-miRNA-10b-5p and serum miRNA-10b-5p.
